# Role of Oral Rabies Vaccines in the Elimination of Dog-Mediated Human Rabies Deaths

**DOI:** 10.3201/eid2612.201266

**Published:** 2020-12

**Authors:** Ryan M. Wallace, Florence Cliquet, Christine Fehlner-Gardiner, Anthony R. Fooks, Claude T. Sabeta, Alvaro Aguilar Setién, Changchun Tu, Vlad Vuta, Boris Yakobson, Dong-Kun Yang, Gideon Brückner, Conrad M. Freuling, Lea Knopf, Artem Metlin, Patricia Pozzetti, Pebi Purwo Suseno, Sean V. Shadomy, Gregorio Torres, Marco Antonio Natal Vigilato, Bernadette Abela-Ridder, Thomas Müller

**Affiliations:** Centers for Disease Control and Prevention, Atlanta, Georgia, USA (R.M. Wallace, S.V. Shadomy);; Agency for Food, Environmental, and Occupational Health and Safety, Malzéville, France (F. Cliquet);; Canadian Food Inspection Agency, Ottawa, Ontario, Canada (C. Fehlner-Gardiner);; Animal and Plant Health Agency, Weybridge, UK (A.R. Fooks);; Onderstepoort Veterinary Institute, Pretoria, South Africa (C.T. Sabeta);; Unidad de Investigación Médica en Inmunología, Coordinación de Investigación en Salud, Instituto Mexicano del Seguro Social (IMSS), Mexico City, Mexico (A.A. Setién);; Academy of Agricultural Sciences, Changchun, China (C. Tu);; Institute for Diagnosis and Animal Health, Faculty of Veterinary Medicine, Bucharest, Romania (V. Vuta);; Kimron Veterinary Institute, Beit Dagan, Israel (B. Yakobson);; Animal and Plant Quarantine Agency, Gimcheong-si, South Korea (D.-K. Yang);; World Organisation for Animal Health, Paris, France (G. Brückner, P. Pozzetti, G. Torres);; Friedrich-Loeffler-Institut, Greifswald-Insel Riems, Germany (C.M. Freuling, T. Müller);; World Health Organization, Geneva, Switzerland (L. Knopf, B. Abela-Ridder);; Federal Centre for Animal Health, Vladimir, Russia (A. Metlin);; Ministry of Agriculture, Jakarta, Indonesia (P. Purwo Suseno);; Food and Agriculture Organization of the United Nations, Rome, Italy (S.V. Shadomy);; Pan American Health Organization, Rio de Janeiro, Brazil (M.A.N. Vigilato)

**Keywords:** rabies, zoonotic, disease control, viruses, zoonoses, dogs, vaccine-preventable diseases

## Abstract

Domestic dogs are responsible for nearly all the »59,000 global human rabies deaths that occur annually. Numerous control measures have been successful at eliminating dog-mediated human rabies deaths in upper-income countries, including dog population management, parenteral dog vaccination programs, access to human rabies vaccines, and education programs for bite prevention and wound treatment. Implementing these techniques in resource-poor settings can be challenging; perhaps the greatest challenge is maintaining adequate herd immunity in free-roaming dog populations. Oral rabies vaccines have been a cornerstone in rabies virus elimination from wildlife populations; however, oral vaccines have never been effectively used to control dog-mediated rabies. Here, we convey the perspectives of the World Organisation for Animal Health Rabies Reference Laboratory Directors, the World Organisation for Animal Health expert committee on dog rabies control, and World Health Organization regarding the role of oral vaccines for dogs. We also issue recommendations for overcoming hesitations to expedited field use of appropriate oral vaccines.

Since the advent of Pasteur’s germ theory and the general acceptance that infectious diseases do not develop spontaneously, humankind has strived to reduce and eliminate pathogens that pose a serious public health threat. Incorporating routine vaccinations to control human diseases such as pneumonia, diarrhea, pertussis, measles, and polio contributed to the prevention of >10 million human deaths during 2010–2015 (*1*). Intensive global efforts torward disease eradication have focused on only a few diseases, including Guinea worm disease (dracunculiasis) (>99% reduction in human cases), smallpox (eradicated in 1980), rinderpest (eradicated in 2011), polio (99% reduction in human cases), and lymphatic filariasis (73% reduction in human cases) (*2*–*6*). Those disease eradication efforts have focused on pathogens that are host-restricted or affect only a single host. In 2015, the world called for action by setting a goal of zero human dog-mediated rabies deaths by 2030 worldwide. In 2018, the World Health Organization (WHO), the World Organisation for Animal Health (OIE), the Food and Agriculture Organization of the United Nations, and the Global Alliance for Rabies Control launched the Global Strategic Plan for global elimination of dog-mediated human rabies deaths by 2030, which represents the first major effort to eliminate a classical zoonosis and poses unique challenges not encountered during prior disease elimination efforts (*7*–*9*).

Among the various rabies reservoir species (*10*,*11*), domestic dogs pose the greatest threat to global public health (*12*,*13*). Dog-mediated rabies is responsible for an estimated 59,000 human deaths annually (95% CI 25,000–159,000) (*14*). Despite the complexities inherent in controlling zoonotic diseases, historical experience has shown that dog-mediated rabies virus elimination is feasible and cost-effective (*15*). Dog-mediated rabies has been eliminated from nearly every high-income country through the implementation of dog vaccination and population management programs (*16*,*17*). Dog rabies control efforts in low- and middle-income countries are estimated to prevent 2.9 million human rabies deaths annually; however, recent examples of successful, large-scale dog rabies elimination in low- and middle-income countries are rare and largely limited to Latin America (*14*,*18*–*22*). In recent decades, vaccination efforts have stagnated in many countries because of scarce funding for animal health sector elimination initiatives and perceived barriers to effectively vaccinating high-risk dog populations (*8*,*23*).

A great paradox exists in the field of global rabies elimination: oral rabies vaccination (ORV) is the main component of elimination of rabies from wildlife populations, which cause only modest human deaths (*24*–*27*), whereas ORV is not used to complement parenteral vaccination for elimination of rabies in dog populations (*28*), which are responsible for more human deaths than any other single zoonotic pathogen. At present, parenteral vaccination is the only approach used for addressing dog-mediated rabies at-scale, despite frequent publications and field reports of the inadequacies of this approach among important subpopulations of susceptible dogs (Table 1) (*39*).

**Table 1:** Examples from the published literature of vaccination campaigns that have met or failed to meet vaccination targets

Successful vaccination programs				Unsuccessful vaccination programs		
Country	Vaccination coverage, %	Reference		Country	Vaccination coverage, %	Reference
Zambia	80	(*29*)		Chad	19	(*30*)
Mexico	>90	(*31*)		Chad	24	(*32*)
Chad	74	(*30*)		Kenya	29	(*33*)
Thailand	70	(*34*)		Nigeria	17	(*35*)
Bolivia	85	(*36*)		South Africa	56	(*37*)
Tanzania	68	(*38*)		Tanzania	9	(*38*)

## Rationale for the Consideration of Oral Vaccines for Dog Vaccination Campaigns

Elimination of dog-mediated rabies from high-income countries was achieved through parenteral vaccination of dogs at fixed locations (e.g., veterinary clinics and fixed community vaccination posts) and has been associated with higher levels of logistical, political, and economic development. In many rabies-endemic countries, the logistical infrastructure is inadequate to support vaccination campaigns capable of reaching adequate herd immunity (estimated at 70%) (*8*). Alternative vaccination methods that overcome these infrastructure deficits, such as capture–vaccinate–release (CVR) and door-to-door vaccination, have been piloted in subnational settings and have shown to be highly effective (*40*–*43*). However, the feasibility of scaling up campaigns that rely upon parenterally focused alternative vaccination methods is now in doubt (*44*,*42*). Parenteral vaccination by CVR techniques has led to tangible reductions in dog-mediated human rabies deaths in areas such as Bali, Indonesia, and Goa, India. However, these programs are relatively small in scale (vaccinating <100,000 dogs per campaign-year). To date, no example of a large-scale, national campaign that relies primarily on the labor-intensive method of CVR exists. The sheer number of vaccinators required to enact the CVR technique at-scale requires a labor pool that does not yet exist in many endemic countries (*8*,*44*). A cadre of an estimated 1.1 million vaccinators would be needed to conduct a national CVR campaign in India (*42*).

In contrast, ORV targets similar dog populations as CVR (i.e., free-roaming) but requires substantially less labor and expertise. Recent studies conducted in Asia and the Americas have shown that although CVR techniques applied to inaccessible dog populations are inefficient (reaching only 10–20 dogs per vaccinator per day), vaccinators using ORV in these same dog populations can far exceed 50 successful vaccinations each day (*42*,*45*,*46*). In settings where alternative vaccination methods are necessary to reach adequate herd immunity, scalability will likely require inclusion of ORV to effectively eliminate dog-mediated rabies.

Although infrastructure is an important component of rabies vaccination, a profusion of other considerations also influence the feasibility of an approach that will be most successful. Whether a population of dogs are accessible for parenteral vaccination depends on cultural, environmental, and economic factors. In most rabies control programs, a positive association exists between dog accessibility and efficiency of vaccinations delivered, as well as cost-effectiveness, predicated on parenteral vaccination. With ORV, this fundamental relationship can change (*46*). Several studies have shown that in areas with low dog accessibility, parenteral vaccination was either ineffective or inefficient, but ORV was able to achieve adequate coverage while remaining a cost-effective public health intervention (*42*,*45*–*48*). Although ORV has been used for >40 years in high-income countries to successfully control and eliminate rabies in wildlife (*24*,*27*), hesitancy to implement ORV as a component of mass dog vaccination campaigns has resulted in a dearth of evidence to argue for the integration, impact, and cost of these vaccines in the context of dog rabies control.

## Factors Contributing to Underutilization of Oral Rabies Vaccines in Dog Vaccination Programs

Decades of debate over the potential role of ORV for dogs has left a confusing landscape of guidance and perspectives (*49*–*53*). Here, we discuss the greatest deterrents to the inclusion of ORV in routine dog vaccination programs and how to encourage their safe and cost-effective use.

### Safety of Oral Rabies Vaccines

**Figure 1:**
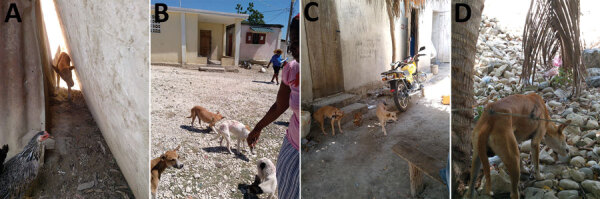
Oral rabies vaccines can be helpful in vaccinating dog populations where traditional parenteral methods have failed to reach adequate vaccination coverages, Haiti. A) A dog hiding behind 2 buildings is vaccinated with an oral rabies vaccine. B) A family with 4 free-roaming dogs watches as they ingest an oral rabies vaccine bait. C) Dogs can be protective of puppies, so oral rabies vaccines provide a safer method to vaccinators and reduce the risk of bites during parenteral vaccination. D) Fences and other barriers can make difficult tasks for parenteral vaccinators. A dog is vaccinated through a barbed wire fence with an oral rabies vaccine.

**Figure 2:**
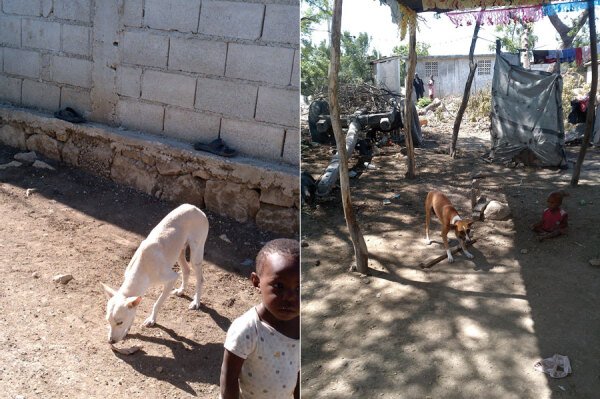
Dogs receiving oral rabies vaccines have the potential to expose community members, particularly children, Haiti. Oral rabies vaccines must be safe for dogs as well as the humans and animals that live near dogs. Children particularly are at risk for exposure to oral rabies vaccines through bites and licks from recently vaccinated dogs or when vaccines are left in the community. The unintended contact with the vaccine can be effectively reduced when a hand-out model (removal of unconsumed or partly ingested baits) is used.

Unintended and long-lasting impacts on humans, as seen with the large-scale field use of oral vaccines for poliovirus and smallpox virus (*54*,*55*), have not been observed with oral rabies virus vaccines (*56*,*57*). Only very few sporadic adverse events in animals or humans, without any epidemiologic impact, have been reported when baits were distributed randomly in the environment (*56*–*63*). Given the close proximity of free-roaming dogs to humans, particularly in urban environments, distributing vaccines through a hand-out model would effectively reduce unintended exposures to vaccine virus compared with environmental distribution of baits (Figures 1, 2).

Thorough safety evaluations are essential before any consideration of field distribution of oral rabies vaccines, including safety for target and major nontarget species, virus dissemination potential, genetic stability, environmental safety, and mode of distribution (Table 2). At least 5 guidelines have been developed to describe the process by which candidate oral vaccines should be evaluated for safety and efficacy (i.e., guidelines issued by OIE, WHO, the European Directorate for the Quality of Medicines, the US Food and Drug Administration, and the US Department of Agriculture Center for Veterinary Biologics) (*49*–*52*,*64*,*65*). These guidelines are highly technical and developed from a regulatory point of view. Publicly available and plain-language vaccine construct safety evaluations have been developed for some veterinary vaccines, such as those released by the European Medicines Agency (https://www.ema.europa.eu/en/medicines/veterinary).

**Table 2:** Summary of recommendations for the evaluation of oral rabies vaccines for vaccine candidates before use in the field

No.	Major categories for assessment of an oral rabies vaccine candidate*
1	Description of the manufacturer
2	Description of the vaccine construct
3	Is the vaccine safe for the target animal?
4	Has safety been assessed for potential non-target animals?
5	Has safety been assessed in nonhuman primates?
6	Does the vaccine elicit an immune response in target animals?
7	Have virulent challenge studies been conducted to assess duration of immunity?
8	Does the vaccine replicate in host tissues and is replicating virus excreted from animals?
9	Is the bait composition attractive to the target animal, and does it convey delivery of the vaccine to the target host-anatomy?
10	Have bait contact rates been described for the bait distribution method you are considering?
11	Has the vaccine been evaluated under field conditions and are storage requirements known?
12	Has an economic cost-benefit assessment been conducted?
13	Is the product currently acknowledged by an international public health agency for field use?
14	Is the product currently licensed in any countries for field use?
15	Is the community supportive of oral rabies vaccination of dogs?
16	Can the responsible authority conduct postvaccination monitoring for persons potentially exposed to the vaccine?
17	Can the responsible authority conduct postvaccination monitoring for vaccine exposures from contact with recently vaccinated dogs?
18	Is there an effective postexposure prophylaxis for humans exposed to the oral rabies vaccine?
19	Can the responsible health authority provide postexposure prophylaxis for persons potentially exposed to the vaccine?

*Adapted from World Health Organization 2007 recommendation on oral rabies vaccine assessment (*51*).

Evaluating of vaccine safety can be a complex and multifaceted process. A standardized safety assessment model was proposed in 2019 by Head et al. (*66*), which describes a method for evaluating the animal and human health impacts of specified vaccine constructs under any potential field-use setting. Increased use of such types of risk assessment tools and dissemination of plain-language safety evaluation results can provide a stronger argument for policy makers to justify the use of oral rabies vaccines. 

To address unfounded concerns related to safety of oral rabies virus vaccines, a few actions are recommended. First, global health agencies should provide guidance on conducting hand-out oral vaccination programs for dogs. Second, global health agencies should provide guidance to policy makers on how to interpret complex safety evaluation studies. Finally, policy-makers should be encouraged to evaluate the animal and human health impacts (beneficial and harmful) from use of ORV as a complement to injectable vaccines in dog vaccination programs.

### Licensure of Oral Rabies Vaccines

Licensure of veterinary products ensures that a national professional regulatory organization has deemed the product safe for the target animal, potential nontarget animals, and humans. Several oral rabies vaccine products are licensed for use in wildlife, yet licensure has not been obtained for these products for use in dogs despite ample studies establishing their safety and efficacy. Vaccine licensure is not a globally consistent process, and licensure in 1 country might not be recognized by others. Licensure, if provided by a highly recognized agency (e.g., US Food and Drug Administration or European Medicines Agency), should translate to wide-ranging use and acceptance of the product globally, because in-country licensure in each rabies-endemic country is neither scientifically necessary nor ethically justified, and therefore unrealistic.

Off-label use of vaccines commonly occurs, especially when broad support from national and international professional organizations (e.g., OIE, WHO, veterinary associations) exists. OIE Reference Laboratories for Rabies, as part of their expertise duties, are available to assist OIE in such a prequalification review process. Only vaccines that have been licensed for wildlife in accordance with international recommendations (*49*–*52*,*64*,*65*) and have shown the highest level of safety and efficacy (based on the provisions for licensing stipulated by OIE) should be considered for prequalification and off-label use in dogs (*67*) (Table 3). Benchmark immunogenicity studies and field trials related to bait acceptance should form the basis for either conditional or full-fledged licensure of oral rabies vaccines for dogs. Given the similar immunologic characteristics of dog populations across countries, results of immunogenicity studies conducted in one country should be considered valid in other countries.

**Table 3:** Landscape analysis of potential oral rabies vaccine candidates

Vaccine name	Manufacturer	Construct	Review status*	Licensure status
V-RG	Boehringer Ingelheim, Germany	Recombinant vaccinia virus	Safe and efficacious	Licensed for wildlife in Europe and USA
ONRAB	Artemis, Canada	Recombinant adenovirus	Safe and efficacious	Licensed for wildlife in Canada
SPBN GASGAS	Ceva, France	Recombinant rabies virus	Safe and efficacious	Licensed for wildlife in Europe
ERA G333	Prokov, Russia	Recombinant rabies virus	Not assessed	Licensed for wildlife in Russia
SAG2	VIRBAC, France	Attenuated rabies virus	Safe and efficacious	Licensed for wildlife in Europe
SAD B19	Ceva, France	Attenuated rabies virus	Low residual pathogenicity	Licensed for wildlife in Europe
SAD Bern	Bioveta, Czech Republic	Attenuated rabies virus	Low residual pathogenicity	Licensed for wildlife in Europe
SAD Clone	Bioveta, Czech Republic	Attenuated rabies virus	Low residual pathogenicity	Licensed for wildlife in Europe
RV-97	FGBI ARRAIH, Russia	Attenuated rabies virus	Low residual pathogenicity	Licensed for wildlife in Russia
KMIEV-94	Institute of Experimental Veterinary, Belarus	Attenuated rabies virus	Not assessed	Licensed for wildlife in Belarus
VRC-RZ2	Kazakhstan	Attenuated rabies virus	Not assessed	Licensed for wildlife in Kazakhstan

*Review status refers to safety and efficacy in the target species based on published data.

To overcome barriers to licensure of oral rabies vaccines, several actions are recommended. First, although licensure can be a long, arduous, and expensive process, manufacturers should continue to seek central licensure for use of their products in dogs. Second, OIE should continue its efforts to promote the concept of vaccine regulatory convergence among OIE member countries. Third, although OIE and WHO recognize the need for use of animal vaccines off-label, a prospective approach to validating oral rabies vaccines, such as the WHO vaccine prequalification process, should be developed to provide more confidence in the use of oral rabies vaccines, both in field-trials and integration into mass parenteral vaccination programs. Fourth, prequalification should be a future requirement for any oral rabies vaccine to be used for dogs in projects funded or supervised by the United Against Rabies initiative, thereby creating an incentive for manufacturers to invest into this area. Finally, OIE and WHO should consider developing a global regulatory science agenda for oral rabies vaccines, similar to what is recommended for human vaccines.

### Production Capacity for Oral Rabies Vaccines

No oral rabies vaccine products that are manufactured at a scale that would meaningfully impact the global or regional burden of dog-mediated rabies are commercially available. A lack of demand from customers (national rabies programs and international funders) and a lack of standard bait flavor, size, and composition for dogs were barriers to large-scale production capacities and implementation of ORV of dogs. In recent years, a near-universal bait flavor has been suggested (egg flavor) and compositions that avoid use of plastics and aluminum foil appear safer when ingested by dogs (*68*). Armed with this new knowledge, mass production of a standard oral rabies vaccine bait seems only limited by a lack of demand. However, field studies should continue to assess bait uptake by modifying bait flavor, composition, and shape. To increase demand of oral rabies vaccines and support sustainable production capacity, OIE and the Pan American Health Organization (PAHO) should offer prequalified oral rabies vaccines through their Vaccine Bank (OIE) and Revolving Fund (PAHO) (*69*,*70*).

### Cost of Oral Rabies Vaccines

With no major production of oral rabies vaccines for dogs currently operationalized, the exact cost of these vaccines remains unclear. Costs are expected to be higher than parenteral vaccines ($2–$4 USD per ORV bait compared with $0.30–$1 USD per parenteral vaccine) (*46*). Despite the relatively high cost per oral vaccine dose, studies have shown that inclusion of ORV as a component of a campaign can increase vaccinator efficiency, leading to overall more cost-effective programs compared with persistent undervaccination that might occur with parenteral-only vaccination methods. Although parenteral vaccination remains the preferred route when the dog is accessible, parenteral vaccination complemented by use of ORV for inaccessible dogs can be a cost-effective approach. International organizations, such as United Against Rabies, should acknowledge the need, role, and acceptability of ORV to further promote safe and cost-effective ORV of dogs

### Role of Oral Rabies Vaccine within a Vaccination Program

Vaccination methods should specifically target dog populations that are essential to the rabies virus transmission cycle (Table 4). Although vaccination of well-owned, often-confined dogs can be performed easily and at low cost, undervaccination of the susceptible dog population leads to persistent rabies endemicity and poor cost-effectiveness (*71*). New tools have been developed to evaluate the conditions under which ORV might be a cost-effective complementary tool within a mixed-methods vaccination campaign (*46*,*72*,*73*). Although parenteral vaccination with high-quality rabies vaccines is preferred for populations of dogs that are accessible (*45*,*74*), complementary ORV of inaccessible dogs increases herd immunity. The OIE-endorsed official program for dog-mediated rabies, the OIE Vaccine Bank, and the PAHO Revolving Fund (*69*,*70*) present opportunities to provide vaccination planning tools to recipients and require verification that adequate planning and preparations have been conducted. Donors and operators of vaccine banks should be aware of these tools and work with donation recipients to ensure that campaigns will result in cost-effective interventions. International organizations should advocate for the use of tools that assess the role of ORV in mixed-method vaccination campaigns to increase awareness of the benefits of ORV.

**Table 4:** Considerations for rabies vaccination methods applied to dog populations

Accessibility of the dog population	Central point	Door-to-door	Capture–vaccinate–release	Oral vaccination
Owned, confined dogs	Good coverage	Good coverage	Moderate coverage	Rarely applicable*
Owned, roaming dogs	Moderate coverage	Moderate coverage	Good coverage	Good coverage
Unowned dogs	Poor coverage	Poor coverage	Good coverage	Good coverage
Advantage	Inexpensive	Owners do not have to transport dogs	Expensive and requires trained staff	Easy and targets free roaming dogs
Disadvantage	Low free-roaming dog coverage	Low free-roaming dog coverage	Cost and scalability concerns	Cost, safety, and efficacy concerns

*Parenteral vaccination should be the preferred method when the dog can be brought for vaccination by an owner.

## Conclusion

Although the goal of global elimination of human deaths from dog-mediated rabies by 2030 was just recently established, expanding vaccination programs and access to human vaccines over the past century has led to a 98% reduction in global human rabies deaths (*14*). The remaining 2% are indeed the proverbial “last mile,” and elimination has proven more difficult because of numerous infrastructural, fiscal, and sociodemographic factors. Although the goal of 2030 will require a comprehensive approach to improve surveillance, human postexposure prophylaxis, dog vaccination and dog population management, and awareness programs, the goal will be more feasible if all tools at our disposal are fully used (Table 5). Perhaps the most underused of all tools in the fight against rabies is ORV of dogs. ORV has a vital role as a complementary tool in the global elimination of dog-mediated human rabies deaths, and specific recommended activities should be pursued urgently to promote safe and cost-effective use of ORV.

**Table 5:** Summary of recommendations to promote the safe and effective use of oral rabies vaccine in dogs*

Short-term activities (activities to be accomplished by 2021)
Global health agencies should provide guidance on conducting hand-out vaccination programs for dogs
Global health agencies should provide guidance to policy makers on how to interpret complex safety evaluation studies
Policy makers should be encouraged to evaluate the animal and human health impacts (beneficial and harmful) from use of ORV as a complement to injectable vaccines in dog vaccination programs
International organizations, such as United Against Rabies, should acknowledge the need, role, and acceptability of ORV to further promote safe and cost-effective ORV of dogs
Vaccination programs should be designed using fit-for-purpose methodology, where appropriate methods (or mixed methods) and vaccine constructs are chosen based on characteristics of the dog population and capacities of the vaccination staff
International organizations should advocate for the use of tools that assess the role of ORV in mixed-method vaccination campaigns to increase awareness of the benefits of ORV
Medium-term activities (activities to be accomplished by 2023)
OIE should continue its efforts to promote the concept of vaccine regulatory convergence to OIE member countries.
Although OIE and WHO do recognize the need for use of animal vaccines off-label, a prospective approach to validating oral rabies vaccines, like the WHO vaccine pre-qualification process, should be developed to provide more confidence in the use of oral rabies vaccines, both in field-trials and integration into mass parenteral vaccination programs
Prequalification should be a future requirement for any oral rabies vaccine to be used for dogs in projects funded or supervised by the United Against Rabies initiative, thereby creating an incentive for manufacturers to invest into this area.
Benchmark immunogenicity studies and field trials should be conducted in several countries representative of regions where dog-mediated rabies is endemic as they are considered crucial to demonstrate the fitness for purpose of oral rabies vaccination as a supplementary tool.
OIE and PAHO should offer these vaccines through their vaccine bank (OIE) and Revolving Fund (PAHO)
Long-term activities (activities to be accomplished by 2025)
Although licensure can be a long, arduous, and expensive process, manufacturers should continue to seek central licensure for use of their products in dogs.
OIE and WHO should consider developing a global regulatory science agenda for oral rabies vaccines, similar to what is recommended for human vaccines

*OIE, World Organisation for Animal Health; ORV, oral rabies vaccination; WHO, World Health Organization; PAHO, Pan American Health Organization.
